# Clinical Effects of Yoga and Meditational Practices on the Holistic Health of Chronic Kidney Disease Patients: A Systematic Review

**DOI:** 10.7759/cureus.57546

**Published:** 2024-04-03

**Authors:** Shikha Gautam, U.V. Kiran

**Affiliations:** 1 Human Development and Family Studies, School of Home Science, Babasaheb Bhimrao Ambedkar University, Lucknow, IND

**Keywords:** quality of life, relaxation techniques, mindfulness meditation, complementary therapies, holistic health, meditation practices, yoga, chronic kidney disease, clinical effect of yoga and meditation, chronic kidney disease (ckd)

## Abstract

As the world accelerates, sedentary and unhealthy lifestyles have an increasingly negative impact on human physical and emotional well-being. Millions of people globally are thought to have chronic kidney disease (CKD), which is frequently brought on by diabetes, hypertension, and glomerulonephritis. Over time, the illness gets worse and eventually results in irreversible renal failure. A person's life can be seriously affected by CKD in many different ways, including emotionally, socially, physically, and financially. Apart from physiological manifestations like anemia, discomfort, and exhaustion, CKD can also result in psychological problems like anxiety and depression, which can impair one's overall standard of life. Numerous studies have demonstrated the beneficial effects of yoga and meditation on people with chronic renal disease, enhancing their general health and quality of life. Because of therapeutic limitations, familial pressures, financial restraints, and symptoms of end-stage kidney disease, people with CKD frequently experience stress and anxiety. By reducing stress and anxiety, yoga and meditation can help individuals with chronic conditions maintain their health and improve their overall well-being. Recent research has found that yoga can improve blood pressure, sympathetic activity, and basal metabolic rate as well as reduce blood pressure and blood sugar levels by balancing the autonomic nervous system. Furthermore, studies have demonstrated that yoga helps CKD patients live healthier lives by lowering stress, anxiety, and sadness. Healthcare professionals can help patients with chronic renal disease manage their symptoms and enhance their general health and well-being by adding yoga and meditation into their treatment regimens. Modifying lifestyle is essential for both the prevention and treatment of chronic renal disease. CKD often co-occurs with other age-related and sedentary lifestyles and poor diet-related chronic conditions. The dearth of targeted treatment for a large percentage of CKD patients led to the investigation of the therapeutic applications of yoga and meditation in this study. These affordable, non-invasive therapies provide a comprehensive approach to controlling CKD, benefiting both healthy individuals and those with CKD in terms of their physical and mental well-being.

## Introduction and background

The World Health Organization estimates that every year, illnesses pertaining to the kidneys and urinary system affect about one million people globally. The two main risk factors for chronic kidney disease (CKD), which is becoming acknowledged as a serious public health concern in India, are hypertension and diabetes. These days, improper lifestyle choices brought on by things like stress, poor diet, and insufficient exercise are the main causes of kidney disease. Patients receiving dialysis and those with chronic renal illnesses may benefit greatly from adopting lifestyle changes centered around yoga and meditation. Various forms of yoga, including controlled breathing techniques (pranayama), physical postures (asanas), hand gestures (mudras), yogic relaxation (Yoga Nidra), and other activities can contribute to improved health outcomes. Meditation practices involve employing a range of techniques to influence the parasympathetic nervous system, leading to calm and relaxed minds. Yoga and meditation originated in India almost 2000 years ago with the goal of fostering human and spiritual development, and they are known to offer multiple health benefits [1].

According to studies, including yoga in one's routine can help patients with chronic illnesses live better because it lessens the possibility of heart disease, mental health problems, and physical dysfunction. Eda (2014) reports that those with chronic diseases like chronic obstructive pulmonary disease, heart disease, breast cancer, obesity, and diabetes mellitus can benefit from yoga. They have been observed to enhance functionality and reduce levels of distress. Regular dialysis patients often suffer from negative side effects, such as an increased risk of cardiovascular disease, functional loss, restlessness, difficulty sleeping, anxiety, depression, and a decline in overall quality of life in conjunction with health. Regular yoga practice, however, has been demonstrated to have beneficial consequences, such as lowering blood pressure in individuals with hypertension, assisting diabetics to regulate their blood sugar, and lowering the chance of cardiac issues for individuals with heart disease. Therefore, yoga may be useful for the treatment of both primary and secondary CKD. However, further research is needed in this field because of the limited existing literature. This study aimed to investigate how yoga and meditation might improve the general health of patients with ongoing renal disease, including their physical and mental well-being. This study examined various implications, including those related to sleep disturbances, sadness, worry, and physical and mental health [2].

**Figure 1:**
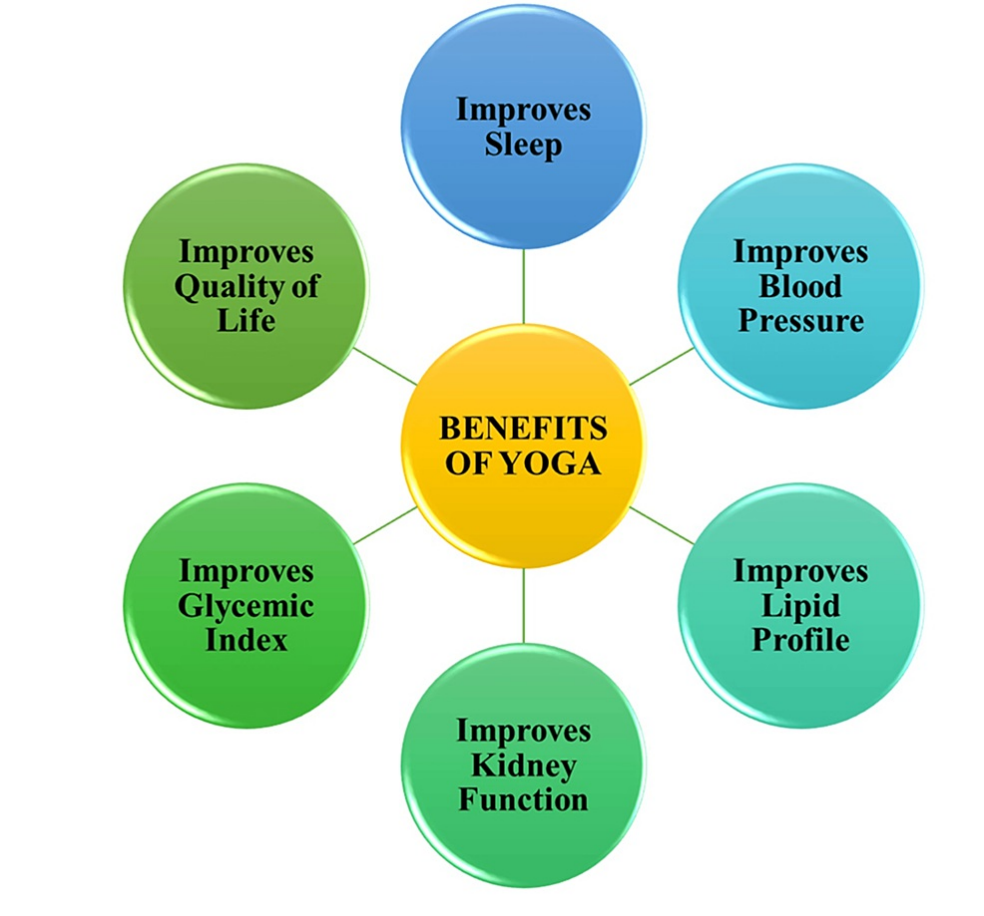
Health benefits of yoga for people with chronic kidney disease. The authors contributed to Figure 1.

CKD encompasses various health outcomes that significantly impact the overall quality of life and exert considerable strain on affected individuals. Common challenges include issues such as poor sleep and appetite, heightened blood pressure and heart rates, and elevated blood sugar and cholesterol levels. The figure presented below provides a concise overview of the manifold benefits derived from engaging in yoga exercises for patients dealing with CKD. These benefits include enhancements in blood pressure, improvements in the lipid profile (specifically cholesterol levels), an overall enhancement in the quality of life, better kidney function, positive impacts on glycemic control indicated by improved blood sugar levels, and an amelioration in sleep quality (Figure 1).

**Figure 2:**
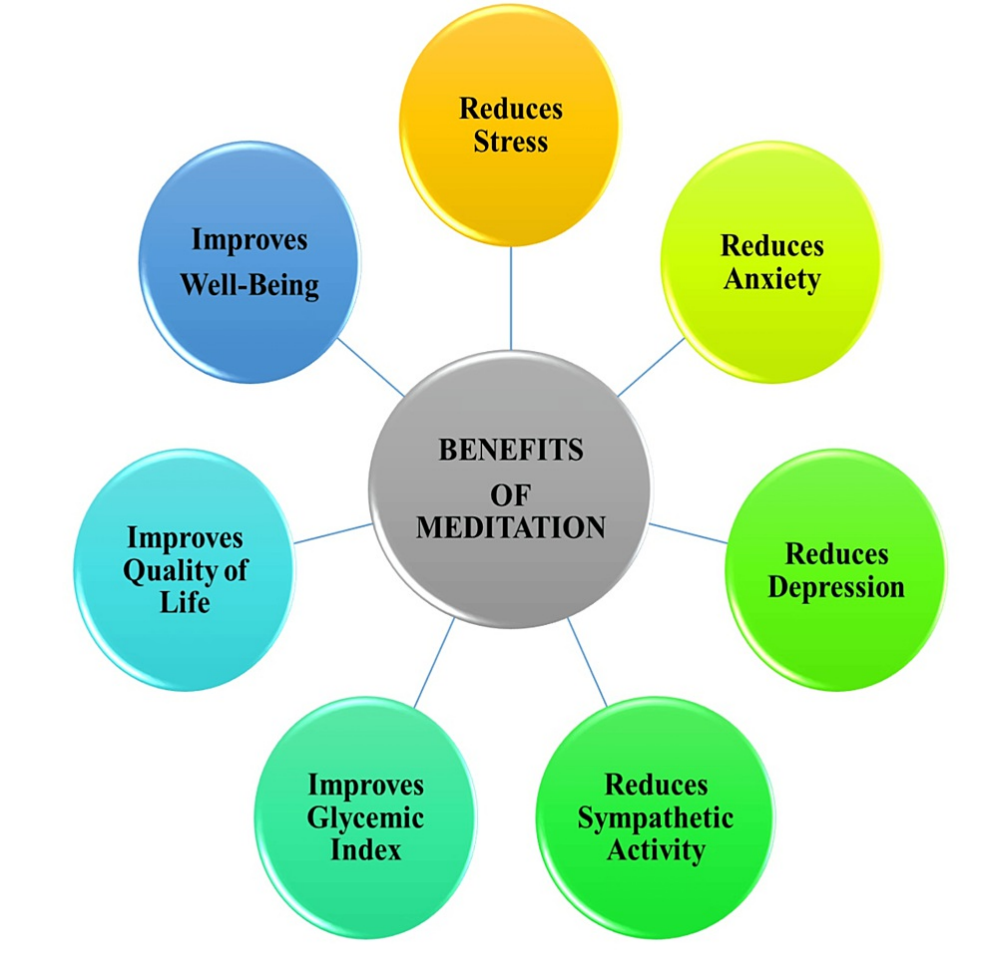
Health benefits of meditation for people with chronic kidney disease. The authors contributed to Figure 2.

A multitude of physiological consequences are associated with CKD, and these consequences have a significant impact on the psychological state of those affected. Typical difficulties include elevated stress, anxiety, and sadness, reduced well-being, and a general decline in life quality. The impact of reduced kidney function extends to the brain, contributing to various mental health issues. Addressing these challenges effectively, without the necessity for additional medical treatments, can be achieved through the practice of meditation. The figure below illustrates the numerous and diverse benefits that meditation can offer to individuals dealing with CKD. These benefits include the reduction of stress, anxiety, and depression through the modulation of sympathetic activity (Figure 2).

## Review

Materials and methods

**Figure 3:**
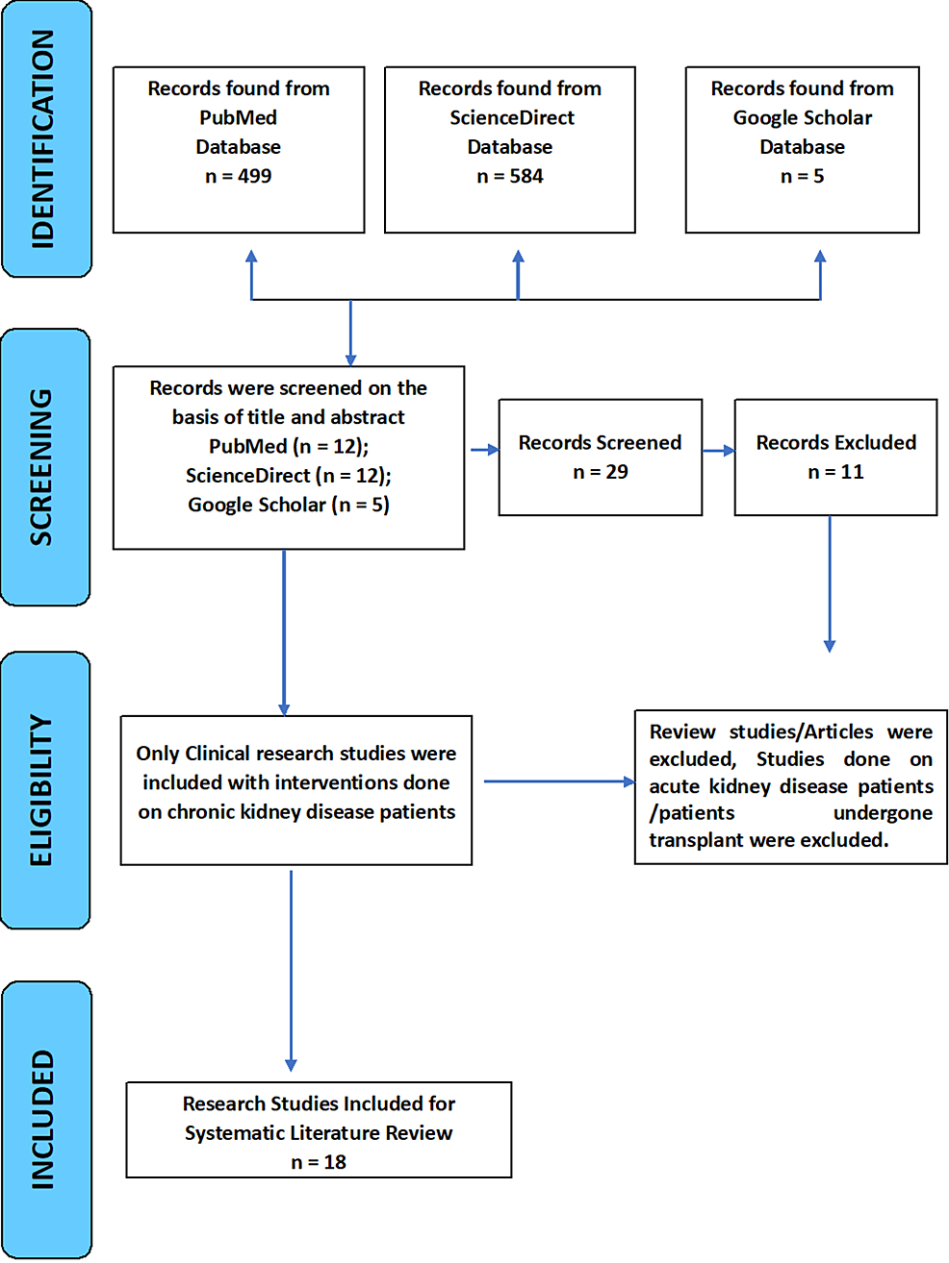
PRISMA flow diagram. PRISMA - Preferred Reporting Items for Systematic Reviews and Meta-Analyses.

Utilizing the Preferred Reporting Items for Systematic Reviews and Meta-Analyses (PRISMA) criteria, we searched extensively on PubMed, Science Direct, and Google Scholar to identify studies that evaluate the clinical effects of yoga therapy and meditation practices on the general health of people with CKD (Figure 3).

Search Strategy

We employed the following search strategy on PubMed to identify relevant articles on the clinical effects of yoga therapy and meditational practices on the overall health of CKD patients. Initially, we identified keywords and used them with Boolean operators, i.e., "clinical effects" and "yoga" or "meditation" and "chronic kidney disease patients."

Inclusion and Exclusion Criteria

The criteria and description to thoroughly investigate relevant research studies related to the review are as per Table 1: criterion 1, only clinical/research studies were included; criterion 2, only those studies were included in which clinical effects of yoga and meditation were measured; criteria 3 and 4, studies done only on CKD patients were included in which we excluded acute kidney disease patients/patients who underwent renal transplant surgery, and patients suffering from other illnesses (Table 1).

**Table 1:** Criteria and description for systematic literature review.

Criteria	Description
Criterion 1	Only clinical/research studies were included.
Criterion 2	Clinical effects of yoga and meditation practices based on intervention were only included.
Criterion 3	Studies done on only chronic kidney disease patients were included.
Criterion 4	Review studies/articles were excluded, and studies done on acute kidney disease patients/patients who had undergone transplants were excluded.

Results

This analysis exclusively considered studies that satisfied the specified inclusion criteria. The studies included a participant range of 10 to 150 individuals, and the intervention duration varied from eight to 24 weeks. The yoga interventions exhibited diversity in terms of length, frequency, and style. The outcome measures, which exhibited diversity, included parameters such as blood pressure, anxiety, depression, and quality of life. Additionally, biochemical indicators like blood nitrogen, creatinine, and glomerular filtration rate were considered. The meditation interventions predominantly concentrated on relaxation techniques and mindfulness meditation.

Mindfulness Meditation

Many people with kidney disease endure stress and anxiety due to the difficulties of end-stage kidney disease (ESKD), which include symptoms, treatment constraints, and different social, economic, and familial obligations. Studies have shown that meditation, which is well-known for its ability to reduce stress and anxiety, can improve the quality of life and possibly increase the longevity of people who are managing chronic diseases. Although there are benefits of incorporating meditation into the dialysis routine for patients with ESKD, its integration is currently limited. A study conducted by Park et al. found that guided meditation had a positive impact on stability, self-confidence, mental clarity, anger management, vitality, enthusiasm, motivation, and self-reflection [3].

There is currently not much data available regarding how Yoga Nidra-guided meditation affects the well-being of patients receiving hemodialysis for chronic renal disease. On the other hand, significant improvements in perceived stress levels as determined by the Perceived Stress Scale point to a possible improvement in kidney disease-related quality of life (KDQOL). Because guided meditation is easy to use and has a high acceptance and compliance rate, it has been shown to be a useful method for improving a variety of areas of well-being and overall quality of life. For individuals undergoing maintenance hemodialysis because of chronic kidney disease, incorporating guided meditation into their care could become a standard practice, potentially improving their quality of life. Advocating this approach may improve patient outcomes. Guided meditation is well suited for CKD patients on dialysis because of its user-friendly nature and positive reception. Further research is necessary to fully comprehend the potential benefits of meditation interventions for improving health outcomes and coping with disease-related challenges. This study should include active control conditions and a larger sample size.

Sleep Disorders

A major problem for those with CKD is sleep difficulties. People with CKD typically report sleep disturbances, and their sleep quality and quantity frequently diminish even in the early stages of the disease. Between 45% and 80% of patients with end-stage renal disease (ESRD) struggle with sleep-related problems; nearly half of individuals with CKD have these problems at the beginning of their illness [4]. Compared to patients receiving continuous ambulatory peritoneal dialysis (PD), patients receiving automated PD appear to have less severe sleep-related breathing disorders (SBD) [5]. There appears to be a decreased incidence of sleep-related issues after kidney transplantation [6].

Various research studies investigated the potential use of medications to enhance sleep quality in individuals with kidney diseases. Clinical settings employ various strategies such as acupressure, exercise, meditation, and medication to improve sleep quality. Evidence regarding the effectiveness of acupressure in improving sleep quality compared to sham acupressure is inconclusive due to the low certainty of the data. Hence, future studies focusing on sleep therapy for individuals with CKD should be prioritized.

Intradialytic Hypotension

In a specific study, laughter yoga (LY) was implemented in an outpatient dialysis center to alleviate intradialytic hypotension. LY is a group exercise that originated in India in 1995 and consists of breathing techniques and simulated laughter. Exercises that encourage smiling and laughing are included, as well as clapping, arm and leg motions, breathing techniques, and light neck and shoulder stretches [7,8]. A study by Paul Bennett at Deakin University looked into the application of intradialytic LY for hemodialysis patients in 2015. This study showed that LY is beneficial for patients with ESRD receiving dialysis. Laughing provides a healthy diversion from the emotionally taxing components of the illness. In addition to deep breathing, clapping, arm and leg stretches, moderate neck and shoulder stretches, and motions that promote smiling and laughing, LY incorporates group breathing exercises and simulated laughter. This study showed positive effects on well-being, including increased energy, a positive outlook, spontaneous laughter, optimism, stress reduction, and improved physical and mental relaxation.

Blood Pressure

When it comes to lowering blood pressure, mindfulness meditation has demonstrated potential for those suffering from persistent CKD, a condition marked by a progressive loss of renal function and increased cardiovascular risk. These disorders are primarily caused by the sympathetic nervous system, and while the creation of sympatholytic medications is crucial, safe and efficient complementary or alternative therapies are also required. Mindfulness meditation, which is a noninvasive and secure approach, offers potential physiological benefits for individuals with CKD, including the reduction of hypertension and sympathetic nervous system overactivation. Researchers explored how mindfulness meditation affected patients with CKD in a study that used microneurography to record the first intraneural recording of muscle sympathetic nerve activity and continuous hemodynamic assessment. The findings showed that in hypertensive individuals with chronic renal illness, a single guided mindfulness meditation session rapidly lowered blood pressure and heart rate [3].

Pain, Anxiety, and Depression

Hatha and restorative yoga have the strongest associations with favorable outcomes in treating pain management, sadness, and anxiety, according to a review study's data [9]. Yoga, which originated in India and has over 30 million practitioners worldwide [10], is a physical and spiritual practice. The concept of yoga therapy, which involves using yoga to improve health and well-being, was introduced in a publication in January 2007 [11]. As a safe and efficient means of promoting physical exercise, improving strength, balance, and flexibility, and possibly easing diseases like depression, heart disease, and high blood pressure, yoga is recommended by the national healthcare system in the United Kingdom [12]. Research has shown that yoga can have a favorable impact on a variety of health aspects, including weight loss, autoimmune disorders, immune system conditions, pain syndromes, psychiatric symptoms and disorders, and pregnancy-related conditions [13]. A 2010 analysis of 21 systematic reviews found consistent positive results for depression and reduced risk of heart disease [14].

Attention, Awareness, and Emotion

Meditation has garnered considerable attention as a discipline aimed at enhancing individuals' attentiveness, awareness, and emotional regulation [15]. Incorporating various techniques rooted in mindfulness or contemplative traditions cultivates heightened mental focus and self-awareness. Several recent studies have emphasized the substantial role of meditation in complementary and alternative medicine (CAM) for therapeutic purposes. This observation is supported by research spanning several decades, starting from the 1950s and including clinical investigations initiated in the 1970s [16]. The increasing recognition of meditation's potential benefits for mental and emotional well-being has led to its growing prominence in CAM. Since people are looking for holistic approaches to health and wellness, meditation has become a significant part of complementary and alternative therapies, making up 25% of all cases.

Fasting Lipid Profile

Individuals with CKD commonly experience persistent or worsening lipid abnormalities despite receiving treatment, and they are also at a higher risk of cardiovascular-related mortality and morbidity. In a study led by Dutta and supported by recent research, it was determined how yoga therapy affected the lipid profiles of CKD patients who fasted. The findings of this study, which align with Dutta's methodology and recent studies, suggest that incorporating yoga practice could serve as an innovative complement to traditional cholesterol-lowering medications, effectively reducing lipid levels in individuals with CKD [17]. All patients in the study had normal or low total cholesterol, low-density lipoprotein (LDL), and high-density lipoprotein (HDL) levels; this may be related to the high occurrence of malnutrition in the Indian population. However, a small percentage of patients had raised triglyceride and very low-density lipoprotein (VLDL) levels. This study shows that yoga is a useful drug-free strategy for treating blood dyslipidemia since it significantly reduces the levels of significant lipid markers such as triglycerides, VLDL, and LDL. To confirm these advantages, more research with a larger sample population is necessary.

Sympathetic and Parasympathetic Systems in Meditation

The brain, spinal cord, and peripheral nervous system (which includes the somatic and autonomic systems) make up the central nervous system, which is responsible for controlling a number of physiological processes. While the sympathetic nervous system is in charge of reactive processes, the parasympathetic nervous system is in charge of the "rest and digest" stages. Sympathetic and parasympathetic activity within the autonomic nervous system, which affects physiological processes, including the heart rate, oxygen saturation, and skin conductance response, is frequently assessed using metrics like low-frequency (LF) heart rate variability (HRV) and LF/high-frequency (HF) ratios. As markers of sympathetic and parasympathetic activity, researchers used LF HRV, LF/HF ratios, HF HRV, and skin conductance response (SCR) in a number of randomized controlled studies centered on meditation-based therapies [18,19]. In scientific investigations of meditation techniques, the body's function has received less attention than mental activities, which have historically been the main focus of meditation practices. Nonetheless, as biomarkers for tracking meditative states, recent research has included markers of autonomic nervous system (ANS) activity, such as heart rate (HR), HRV, SCR, respiratory amplitude/rate, and EEG frequencies [18]. A shift not seen in the relaxation group following training was noted in the EEG power within the theta frequency range (3-8 Hz) in the meditation group, according to studies using frontal midline electrodes Fz, FCz, and Cz connected to the anterior cingulate cortex (ACC) [20]. Furthermore, research on integrative body-mind training (IBMT) has demonstrated that incorporating both "bodifulness" and mindfulness into meditation can alter brain physiology and plasticity, impacting the functioning of both the ANS and the CNS [20].

An extensive summary of relevant research studies in our ongoing review emphasizing the importance of incorporating yoga and meditation practices for holistic health benefits in individuals with CKD is presented in Table 2.

**Table 2:** Summary of all the cases outlining the paper title, paper abstract, intervention, and outcomes measured. CKD: chronic kidney disease; IDY: intradialytic yoga; MHD: maintenance hemodialysis; CAM: complementary and alternative medicine; ESRD: end-stage renal disease; HRQOL: health-related quality of life.

Author	Year	Research Study	Abstract	Intervention/s	Outcomes measured
Birdee et al. [1]	2015	Feasibility and safety of intradialysis yoga and education in maintenance hemodialysis patients	This study compared the feasibility and safety of yoga versus kidney education to promote physical activity in patients with end-stage renal disease undergoing maintenance hemodialysis, who are generally less active than healthy individuals.	Feasibility and safety of intradialysis yoga and education	Substantial chances of the yoga intervention's success and absence of unfavorable outcomes.
Park et al. [3]	2014	Mindfulness meditation lowers muscle sympathetic nerve activity and blood pressure in African-American males with chronic kidney disease	Mindfulness meditation may have a short-term positive impact on CKD patients' autonomic and hemodynamic functions.	Mindfulness meditation	Acutely lowers blood pressure and heart rate in African-American males with hypertensive CKD.
Dutta et al. [17]	2020	Effect of yoga therapy on fasting lipid profile in chronic kidney disease: a comparative study	Patients with CKD frequently have abnormal cholesterol levels, and these may continue or get worse even after treatment is started. In this population, cardiovascular mortality and morbidity are still very high.	Yoga therapy	Substantial decrease in lipid profile.
Pandey et al. [21]	2017	Effects of 6 months yoga program on renal functions and quality of life in patients suffering from chronic kidney disease	Patients with CKD can improve their quality of life and renal functioning by participating in a six-month yoga program, which is safe and beneficial as an adjuvant therapy.	Yoga therapy	Improved quality of life and kidney functions of the patients suffering from this disease.
Kothari et al. [22]	2021	Impact of Arham Purushakar meditation on overall well-being of patients with chronic kidney disease on hemodialysis	A useful complementary therapy for people on hemodialysis is meditation.	Arham Purushakar meditation	Improved mental health and well-being of people with CKD.
Kalita [23]	2021	A pilot study on effect of Yoga-Nidra programme on depression, anxiety, and stress among patients with chronic kidney disease receiving haemodialysis	One especially effective relaxation method for individuals undergoing hemodialysis is Yoga-Nidra.	Yoga-Nidra program	Improved various aspects of mental health.
Bantornwan et al. [24]	2014	Role of meditation in reducing sympathetic hyperactivity and improving quality of life in lupus nephritis patients with chronic kidney disease	Sympathetic hyperactivity, which leads to perishing, is a common characteristic of systemic lupus erythematosus (SLE). According to some physiological research, meditation may lessen this autonomic dysfunction.	Meditation	Meditation helps patients with CKD and lupus nephritis by lowering sympathetic overactivity and enhancing quality of life.
KauricKlein [25]	2019	Effect of yoga on physical and psychological outcomes in patients on chronic hemodialysis	Patients on chronic hemodialysis exhibited improvements in a number of outcomes when they practiced yoga.	Intradialysis yoga	Improved physical and psychological outcomes in patients.
Yurtkuran et al. [26]	2007	A modified yoga-based exercise program in hemodialysis patients: a randomized controlled study	For patients with end-stage renal illness, an optimized yoga-based rehabilitation program provides an additional safe and efficient clinical treatment option.	A modified yoga-based exercise program	Yoga was found to be an effective clinical modality for various kidney-related health outcomes such as blood urea and creatinine, erythrocyte, hemoglobin, etc.
Balaji et al. [27]	2012	Physiological effects of yogic practices and transcendental meditation in health and disease	Yoga lowered blood pressure, body mass index, diabetes, cardiovascular risk, and improved breathing and cognitive function.	Yogic practices and transcendental meditation (TM)	Yogic practices and transcendental meditation improved cognitive function, respiratory system, cardiovascular health, blood pressure, and glycemic index.
Igarashi et al. [28]	2021	The effects of a short-term meditation-based mindfulness protocol in patients receiving hemodialysis	In patients on chronic hemodialysis, meditation increased levels of self-compassion and serum phosphorus.	Short-term meditation protocol	Improved various depressive symptoms and enhanced compassion and mindfulness.
Markell [29]	2005	Potential benefits of complementary medicine modalities in patients with chronic kidney disease	There is growing evidence that a number of complementary and alternative medicine approaches help individuals with long-term illnesses.		Improved disease progression, bone health, disease, cardiovascular health, anxiety, depression, and fatigue. It had hepatoprotective factors and treatment of uremic bruising.
Vaishnav et al. [30]	2022	Study of effect of guided meditation on quality of life in patients of end stage renal disease (ESRD) on maintenance hemodialysis – a randomised controlled trial	Guided meditation dramatically increased the qualitative well-being aspects of happiness, excitement, inspiration, activeness, alertness, awareness, stability, self-confidence, mental clarity, anger management, and self-reflection among CKD patients.	Guided meditation	All measures of overall physical well-being and happiness showed statistically significant differences.
Bennett et al. [31]	2015	Intradialytic laughter yoga therapy for haemodialysis patients: a pre-post intervention feasibility study	This study offers assurance that laughter yoga is a low-risk, effective kind of intradialytic exercise that can be used with patients on dialysis.	Intradialytic laughter yoga	It improves mood and decreases anxiety.
Herron et al. [32]	2023	A pilot randomized trial of intradialysis yoga for patients with end-stage kidney disease	To assess the impact of an IDY on MHD patients' quality of life, self-efficacy, and physical performance, randomized clinical pilot research is being considered.	Intradialytic yoga	Improves anxiety, sadness, and chronic pain among maintenance hemodialysis patients.
Shamsuddin et al. [33]	2019	Pattern of complementary and alternative medicine (CAM) use among patients with chronic kidney disease	CAM therapies that help patients with CKD include natural goods like vitamins, minerals, and herbs; also, mind-body techniques like yoga, meditation, and music therapy are beneficial.	Complementary and alternative medicine (CAM)	Mixed findings in the literature about the pros and cons of CAM.
Alhawatmeh et al. [34]	2022	Effects of mindfulness meditation on trait mindfulness, perceived stress, emotion regulation, and quality of life in hemodialysis patients: a randomized controlled trial	Hemodialysis has been identified as a stressor that lowers health-related quality of life, along with the indications and symptoms of end-stage renal disease. Numerous physical and psychiatric disorders have been demonstrated to benefit greatly from mindfulness meditation.	Mindfulness meditation	Manages stress and health, decreases perceived stress, and increases trait mindfulness, emotion regulation, and quality of life.
Gross et al. [35]	2017	Telephone-adapted mindfulness-based stress reduction (tMBSR) for patients awaiting kidney transplantation	The quality of life related to health declines as physiological and psychological stressors increase in patients with progressive renal disease. When it comes to improving ESRD patients' mental HRQOL, tMBSR is more beneficial than support.	Telephone-adapted mindfulness-based stress reduction (tMBSR)	Anxiety reduced

The aggregated findings of this research indicate an increasing amount of data demonstrating the value of yoga and meditation as supplemental therapies for the treatment of CKD. These practices are not only gaining recognition but are increasingly recognized as integral components of a holistic healthcare approach for CKD patients. The synthesis of research studies emphasizes the diverse advantages associated with implementing yoga and meditation in the CKD population, extending beyond physical health to include mental, emotional, and spiritual well-being. This holistic approach addresses various dimensions of an individual's health, contributing to an enhanced quality of life. The studies featured in the table underscore the positive impact of yoga and meditation on crucial parameters like stress reduction, blood pressure regulation, and overall mental health in CKD patients. Additionally, the evidence suggests a potential role in slowing down CKD progression and alleviating associated symptoms.

Therefore, the synthesis of past research unequivocally supports the idea that integrating yoga and meditation into the care regimen for CKD patients holds great promise for fostering comprehensive health improvements. As we move forward, it is crucial for healthcare professionals to view these mind-body practices as valuable complements to conventional medical approaches, aligning with the current trend of patient-centered care and emphasizing the interconnected dimensions of health in chronic condition management.

## Conclusions

Research suggests that patients with CKD can derive significant benefits from practicing yoga and meditation. These practices can lead to improvements in overall well-being, stress reduction, and physical functioning. Furthermore, they can be of great help to individuals dealing with various health issues. Yoga therapy has been shown in numerous studies to positively affect a number of health indices, including blood pressure, mental health, and quality of sleep. Additionally, multiple studies have found that creatinine levels, blood nitrogen levels, and glomerular filtration rates can be improved. By consistently engaging in yoga and meditation, individuals with kidney disease can experience an enhanced quality of life and better health outcomes. However, it is crucial to consult a doctor before starting any exercise program including yoga sessions. Numerous medical and psychological disorders, such as hypertension, diabetes, obesity, cognitive decline, stress, anxiety, and depression, are frequently present in conjunction with CKD. These conditions are frequently linked to a sedentary lifestyle and can exacerbate CKD progression. To address these issues, integrative or functional medicine practitioners frequently recommend incorporating mind-body interventions into holistic treatment plans. For instance, mindfulness meditation has shown promise in improving the psychological well-being of patients with CKD by reducing feelings of worry and sadness. To completely comprehend the processes via which yoga and meditation can help people with CKD and to ascertain the ideal frequency and duration of these interventions, more research is necessary. While early evidence indicates that yoga and meditation may be beneficial in managing CKD, further studies are needed to completely understand their clinical relevance and efficacy in this population. Overall, the integration of yoga therapy and meditation practices may offer a valuable approach to enhancing the health and well-being of patients with CKD.
